# Algorithmic approach in the diagnosis of pediatric uveitis

**DOI:** 10.1186/s12969-024-00962-8

**Published:** 2024-02-14

**Authors:** Farhad Salehzadeh

**Affiliations:** Pediatric Department, Bouali Children’s Hospital, Ardabil University of Medical Sciences (ARUMS), Ardabil, Iran

Uveitis is defined as an intraocular inflammation involving the iris, ciliary body, or choroid [1]. Uveitis is caused by disorders of diverse etiologies including wide spectrum of infectious and non-infectious causes. These entities can be categorized into three groups based on their etiologies: infectious, non-infectious, and masquerade syndrome [2].

“Infectious uveitis” including viruses, bacteria, fungi, parasites, and mycobacteria, may directly infect intraocular tissues or trigger host immune response that led to ocular inflammation.

“Non-infectious uveitis” refers to uveitis resulting from immune-mediated inflammation, which may be associated with autoimmune diseases or may be a solitary ocular disease. Trauma of the eyeball, intraocular procedures, or medications may also induce uveitis [1, 2].

Masquerade syndrome refers to lymphoma, intraocular foreign body, retinal detachment, or other causes that may mimic intraocular inflammation; it should always be considered and ruled out before making the diagnosis [3].

Uveitis in the pediatric differs from adult uveitis in that it is commonly asymptomatic but can become chronic and cause damage to ocular structures. The diagnosis may be delayed for many reasons, including the difficulties in communicating with and examining young children. Uveitis morbidities in pediatric patients include cataract, glaucoma, and amblyopia [4].

The International Uveitis Study Group (IUSG) and Standardization of Uveitis Nomenclature (SUN) criteria allow ophthalmologists to classify uveitis for research and clinical purposes. The SUN criteria divide uveitis into anterior uveitis (iris and the anterior ciliary body), intermediate uveitis (posterior ciliary body and vitreous), posterior uveitis (retina and/or choroid), and panuveitis (all structures affected) [5].

According to the (SUN) criteria, regarding timing, uveitis is classified as acute (duration of less than 6 weeks), recurrent (repeated episodes of uveitis separated by periods of inactivity ≥ 3 months in duration without treatment), and chronic (persistent uveitis characterized by relapse in less than 3 months after discontinuation of therapy [4].

Most cases of pediatric uveitis are non-infectious (67.2– 93.8%) [6]. Most studies report JIA as the main underlying systemic disease associated with childhood non-infectious uveitis [6].

Toxoplasmosis is the most common form of infectious uveitis in most studies [7]., Toxocariasis is the second infectious cause of uveitis [4]. In Europe, poststreptococcal uveitis accounts for 2.5–10.6% of pediatric cases [3].

Etiological diagnosis of uveitis begins with the first step of taking history followed by systemic and ocular examination, subsequently there is a long list of differential diagnosis to rule out or rule in the possible etiology. With lack of algorithmic approach laboratory investigation has its own challenging, and other subspecialty consultation may be required.

The present figure (Fig. 1) defines a practical algorithmic approach in diagnosis of pediatric uveitis. Algorithms solve problem by showing the step-by-step evaluation. Steps in building of this algorithm include the following criterion: location as anterior or posterior or intermediate, and unilateral or bilateral findings and the course of disease as an acute, or chronic involvements. If patients have anterior uveitis, then characteristic features as chronic or acute form, alongside with single or both eyes involvement help to focus some limited causes. Same approaches are in chronic or intermediate and posterior uveitis.

**Fig. 1:**
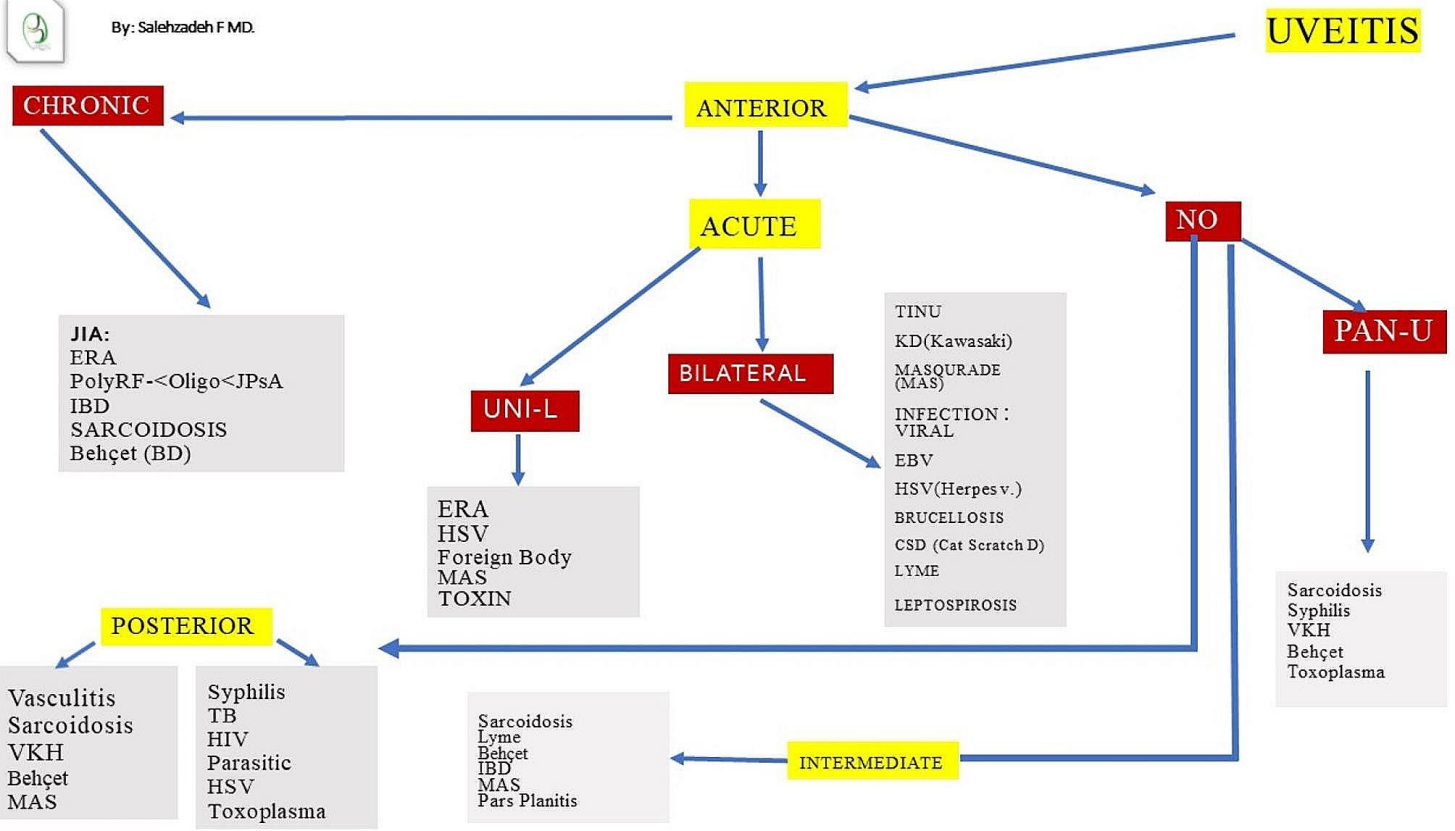
Algorithmic approach in the diagnosis of pediatric uveitis. VKH: Vogt Kayanagi Harada TINU: Tubulointerstitial Nephritis and Uveitis Syndrome MAS: masquerade CSA: Cat scratch disease KD: Kawasaki Disease TB: tuberculosis JIA: Juvenile Idiopathic arthritis BD: Behçet disease IBD: Inflammatory bowel disease ERA: Enthesitis-related arthritis HSV: herpes simplex virus JPsA: juvenile psoriatic arthritis

Although this algorithm may lack some uncommon diseases, PRSI (Pediatric Rheumatology Society of Iran) proposed this algorithmic approach for stepwise diagnostic manner in pediatric uveitis.

## Data Availability

please contact author (Salehzadeh F.) for all data requests.

## Abbreviations

VKH: Vogt Kayanagi Harada
TINU: Tubulointerstitial Nephritis and Uveitis Syndrome
MAS: Masquerade
CSA: Cat scratch disease
KD: Kawasaki Disease
TB: Tuberculosis
JIA: Juvenile Idiopathic arthritis
BD: Beh?et disease
IBD: Inflammatory bowel disease
ERA: Enthesitis-related arthritis
HSV: Herpes simplex virus
JPsA: Juvenile psoriatic arthritis
IUSG: International Uveitis Study Group
SUN: Standardization of Uveitis Nomenclature
